# Long COVID brain fog and muscle pain are associated with longer time to clearance of SARS-CoV-2 RNA from the upper respiratory tract during acute infection

**DOI:** 10.3389/fimmu.2023.1147549

**Published:** 2023-04-28

**Authors:** Annukka A. R. Antar, Tong Yu, Zoe O Demko, Chen Hu, Jeffrey A. Tornheim, Paul W. Blair, David L. Thomas, Yukari C. Manabe

**Affiliations:** ^1^ Department of Medicine, Johns Hopkins University School of Medicine, Baltimore, MD, United States; ^2^ Department of Oncology, Johns Hopkins University School of Medicine, Baltimore, MD, United States; ^3^ Austere environments Consortium for Enhanced Sepsis Outcomes (ACESO), Henry M. Jackson Foundation for the Advancement of Military Medicine, Inc., Bethesda, MD, United States

**Keywords:** COVID-19, long COVID, post-acute COVID-19 syndrome, post-acute sequelae of SARS-CoV-2 infection, brain fog, cognitive dysfunction, pain, reservoir

## Abstract

**Introduction:**

The incidence of long COVID is substantial, even in people with mild to moderate acute COVID-19. The role of early viral kinetics in the subsequent development of long COVID is largely unknown, especially in individuals who were not hospitalized for acute COVID-19.

**Methods:**

Seventy-three non-hospitalized adult participants were enrolled within approximately 48 hours of their first positive SARS-CoV-2 RT-PCR test, and mid-turbinate nasal and saliva samples were collected up to 9 times within the first 45 days after enrollment. Samples were assayed for SARS-CoV-2 using RT-PCR and additional SARS-CoV-2 test results were abstracted from the clinical record. Each participant indicated the presence and severity of 49 long COVID symptoms at 1-, 3-, 6-, 12-, and 18-months post-COVID-19 diagnosis. Time from acute COVID-19 illness onset to SARS-CoV-2 RNA clearance greater or less than 28 days was tested for association with the presence or absence of each of 49 long COVID symptoms at 90+ days from acute COVID-19 symptom onset.

**Results:**

Self-reported brain fog and muscle pain at 90+ days after acute COVID-19 onset were negatively associated with viral RNA clearance within 28 days of acute COVID-19 onset with adjustment for age, sex, BMI ≥ 25, and COVID vaccination status prior to COVID-19 (brain fog: aRR 0.46, 95% CI 0.22-0.95; muscle pain: aRR 0.28, 95% CI 0.08-0.94). Participants reporting higher severity brain fog or muscle pain at 90+ days after acute COVID-19 onset were less likely to have cleared SARS-CoV-2 RNA within 28 days. The acute viral RNA decay trajectories of participants who did and did not later go on to experience brain fog 90+ days after acute COVID-19 onset were distinct.

**Discussion:**

This work indicates that at least two long COVID symptoms - brain fog and muscle pain – at 90+ days from acute COVID-19 onset are specifically associated with prolonged time to clearance of SARS-CoV-2 RNA from the upper respiratory tract during acute COVID-19. This finding provides evidence that delayed immune clearance of SARS-CoV-2 antigen or greater amount or duration of viral antigen burden in the upper respiratory tract during acute COVID-19 are directly linked to long COVID. This work suggests that host-pathogen interactions during the first few weeks after acute COVID-19 onset have an impact on long COVID risk months later.

## 1 Introduction

The incidence of long coronavirus disease (COVID) is substantial, even in people who did not require hospitalization for acute COVID-19 (1). The United States Census Household Pulse Survey of December 2022 estimates that 28% of U.S. adults who ever had COVID-19 experienced long COVID symptoms lasting 3 months or longer (2). Among the most common and debilitating symptoms are pain and a lack of mental clarity or problems with concentration, also called “brain fog” (3, 4). Viral antigen persistence in tissues may be one mechanism of long COVID (5, 6). However, few longitudinal studies with well-characterized viral dynamics in the acute phase have assessed the relationship between viral persistence and long COVID symptoms, especially in people with mild to moderate acute COVID-19 who represent typical disease severity in the general population. Here, we examine the association between time to viral RNA clearance from the upper respiratory tract (URT) during acute COVID-19 and specific clinical symptoms of long COVID at 90 or more days from acute COVID-19 illness onset in a cohort of people diagnosed with symptomatic COVID-19 in the outpatient setting.

## 2 Methods

A convenience sample of non-hospitalized adults with a first positive severe acute respiratory syndrome coronavirus 2 (SARS-CoV-2) PCR test within the previous 48 h from the Johns Hopkins Health System and their household contacts were prospectively enrolled between 21 April 2020 and 28 October 2021 (**Supplementary Figure 1**) (7). Participants provided verbal consent after documentation of understanding, using a consent waiver with an alteration of informed consent, as most were isolating or quarantining at home. The study was conducted in either English or Spanish. This study protocol and verbal consent were approved by the Johns Hopkins University School of Medicine Institutional Review Board.

Mid-turbinate nasal swabs and saliva (URT samples) were collected at 1, 3, 5, 7, 14, 21, and 28 days from enrollment. Some participants had the option of study sampling on days 5, 10, 35, and 42 from enrollment. Mid-turbinate nasal swabs were collected in 600-µl volumes of viral transport media, and 200-µl volumes of saliva were collected. Collections at 1, 3, 5, 7, 10, 14, 21, 35, and 42 days after study enrollment were self-collected into study-provided vials with guidance by phone or video call from the study staff and frozen immediately. Collections at 28 days after study enrollment were performed primarily in-person at a research clinic visit with the study staff guiding or performing the collection. SARS-CoV-2 RT-PCR testing was performed on all samples (Abbott *m*2000 platform, Abbott Park, IL, USA) according to the manufacturer’s instructions as described previously (8). Discordant results from samples collected on the same day (e.g., positive nasal, negative saliva) were recorded as URT positive. The adequacy of self-collected samples was confirmed via RT-PCR for GAPDH human gene expression utilizing a TaqMan gene expression assay for the first 1,338 samples (8). Briefly, eluates from the Abbott *m*2000 were analyzed by the NanoDrop One (Thermo Fisher Scientific, Waltham, MA, USA) to determine total RNA concentration, and all samples were normalized to 1 ng/µl of total RNA prior to analysis for GAPDH. Normalized samples were utilized to synthesize cDNA in 10-µl volumes utilizing Superscript IV RT (Thermo Fisher) according to the manufacturer’s instructions. After cDNA synthesis, samples were assessed for the presence of gapDH utilizing RT-PCR (Applied Biosystems, Foster City, CA, USA) according to the manufacturer’s instructions. Positive reactions were defined as reactions having a cycle threshold value <40. One thousand two hundred twenty-seven of 1,338 self-collected samples were positive for human GAPDH, and all negative reactions started from eluates with <1 µl, indicating insufficient material for NanoDrop analysis or cDNA synthesis. COVID-19 PCR testing results from the clinical electronic medical records were also included in this analysis.

At 1, 3, 6, 12, and 18 months post-COVID-19 diagnosis, participants were asked, “Have you had any of the following symptoms in the past week?” and selected one of the following response options—”Not at all,” “A little bit,” “Somewhat,” “Very much,” or “Quite a bit”—for each of the 49 long COVID symptoms (**Table 1**). Thirty-eight symptoms were derived from the FLU-PRO^©^ Plus (9), and 11 additional symptoms were added based on the frequency of report in a Long COVID Patient-Led Research Collaborative survey with input from the patient-researcher authors (3). Similar to other symptoms, brain fog was assessed with the question “Have you had problems with concentration or ‘brain fog’ in the past week?”.

**Table 1:** List of the 49 long COVID symptoms assessed in surveys at 1, 3, 6, 12, and 18 months from enrollment.

Category	Symptom
Body/systemic	Fever (>100.4°F/>38.0°C or suspected but temperature unknown)
	Low-grade fever (99.0-100.3°F/37.2-37.9°C)
	Chills or shivering
	Felt cold
	Felt hot
	Sweating
	Head congestion
	Constant thirst
	Lack of appetite
	Body aches or pain
	Joint pain
	Muscle pain
	Leg swelling
	Sleeping more than usual
	Trouble sleeping or insomnia
	Weak or tired or fatigued
Neurologic	Headache
	Felt dizzy
	Problems with balance
	Confusion
	Memory problems
	Problems with concentration or “brain fog”
	Seizure
	Hallucinations or lucid dreaming
	Anxiety
	Ringing in the ears
Eye	Teary or watery eyes
	Sore or painful eyes
	Eyes sensitive to light
Nose	Runny or dripping nose
	Congested or stuffy nose
	Sinus pressure
Throat	Scratchy or itchy throat
	Sore or painful throat
	Difficulty swallowing
Chest/respiratory	Trouble breathing/shortness of breath
	Dry or hacking cough
	Wet or loose cough
	Chest congestion
	Chest pain or pressure, chest tightness or burning in the chest
	Heart palpitations; funny or fast heartbeat
Smell/taste	Full or partial loss of taste
	Full or partial loss of smell
Gastrointestinal	Felt nauseous (feeling like you want to throw up)
	Vomiting
	Stomachache
	Diarrhea
Skin	Tingling, numbness, coldness, or other unusual sensation in the skin
	Rash

Participants were asked, “Have you had any of the following symptoms in the past week?” and selected one of the following response options—”Not at all,” “A little bit,” “Somewhat,” “Very much,” or “Quite a bit”—for each symptom.

Time to SARS-CoV-2 RNA clearance was defined as the number of days between acute COVID-19 illness onset (recorded at study enrollment) and the midpoint between a participant’s last positive URT RNA test and the following negative URT PCR test. Time to viral RNA clearance was classified as a binary variable: within or beyond 28 days. This timepoint was chosen because the median viral RNA clearance time obtained from a Kaplan–Meier analysis of participants who completed acute sampling and at least one survey 1+ months from acute COVID-19 illness onset was 27.5 days. Additionally, the last required acute study sampling day was study day 28.

For each of the 49 long COVID symptoms, we estimated the relative risk of reporting that symptom 90+ days from acute COVID-19 onset by time to viral RNA clearance within 28 days using log-binomial regression (10) and adjusting for age, sex, BMI over/under 25, and COVID vaccination status prior to acute COVID-19 (1+ vaccines *vs*. none) (Stata/SE 17, StataCorp, College Station, TX, USA). The maximum severity of each symptom was defined as the highest score from 1 (“Not at all”) to 5 (“Quite a bit”) for that symptom that each participant reported 90 or more days after acute COVID-19 illness onset and was used as a continuous predictor in an adjusted logistic regression model to estimate the association of maximum severity of symptoms in the late post-acute phase and acute viral RNA clearance. We used functional data analysis methods to visualize the curve of the RT-PCR cycle threshold values over time by grouping participants with or without brain fog at 90 or more days from symptom onset.

## 3 Results

Seventy-three participants with symptomatic, PCR-confirmed acute COVID-19, age ≥18, with ≥2 URT samples within 28 days of enrollment, and who completed at least one comprehensive symptom survey ≥90 days from acute COVID-19 onset were included in this analysis (**Supplementary Figure 1**). The participants had a median of 3 study days with a positive PCR test prior to a study day with a negative PCR test, and 75% of the participants had ≥5 study days with PCR tests available for analysis. The median time to viral RNA clearance was 17 days for the 51 participants who had a negative PCR test within 21 days of their last positive PCR test (interquartile ratio, IQR, 14.75-22.75). Twenty-nine (40%) participants met this cohort’s definition of long COVID by reporting in the survey at least once at study months 3, 6, 12, or 18 that they had not returned to their usual pre-COVID health status and also complained of at least one symptom.

The median age of the participants was 52 years (IQR, 42-60), 63% were women, 27% self-reported as non-Hispanic African American, 52% self-reported as non-Hispanic White, and 16% self-reported as Hispanic (**Table 2**). All participants tested positive for COVID-19 in the outpatient setting, and two (2.7%) ultimately required hospitalization during acute COVID-19. Of the participants, 81% were unvaccinated prior to COVID-19, 5% had incomplete primary vaccine series, and 14% had received a complete primary vaccine series prior to COVID-19 (**Table 2**).

**Table 2:** Participants’ demographics, body mass index, and pre-COVID vaccination status.

		Time to viral RNA clearance from the URT >28 days from acute COVID-19 onset (*n* = 30)	Time to viral RNA clearance from the URT ≤28 days from acute COVID-19 onset (*n* = 43)
Age at enrollment, median (IQR), years		54 (46-65)	50 (40-58)
Female, no. (%)		18 (60)	28 (65)
Race and ethnicity, no. (%)	Non-Hispanic African American	9 (30)	11 (26)
	Non-Hispanic White	15 (50)	23 (53)
	Non-Hispanic other	1 (3)	2 (5)
	Hispanic	5 (17)	7 (16)
BMI ≥ 25 kg/m^2^, no. (%)		25 (83)	29 (67)
Prior diagnosis of hypertension, no. (%)		14 (47)	12 (28)
Prior diagnosis of diabetes, no. (%)		7 (23)	6 (14)
Prior diagnosis of anxiety, depression, or bipolar, no. (%)		5 (17)	15 (35)
Median time to viral RNA clearance (IQR), days		74 (37-132)	16 (14-21)
Hospitalized during acute COVID-19, no. (%)		2 (7)	0 (0)
COVID vaccination status prior to COVID-19, no. (%)	No vaccine doses	29 (97)	30 (70)
	Incomplete primary vaccination series	0 (0)	4 (9)
	Complete primary vaccination series	1 (3)	9 (21)

URT, upper respiratory tract encompassing mid-turbinate nasal swab and saliva; BMI, body mass index.

**Figure 1:**
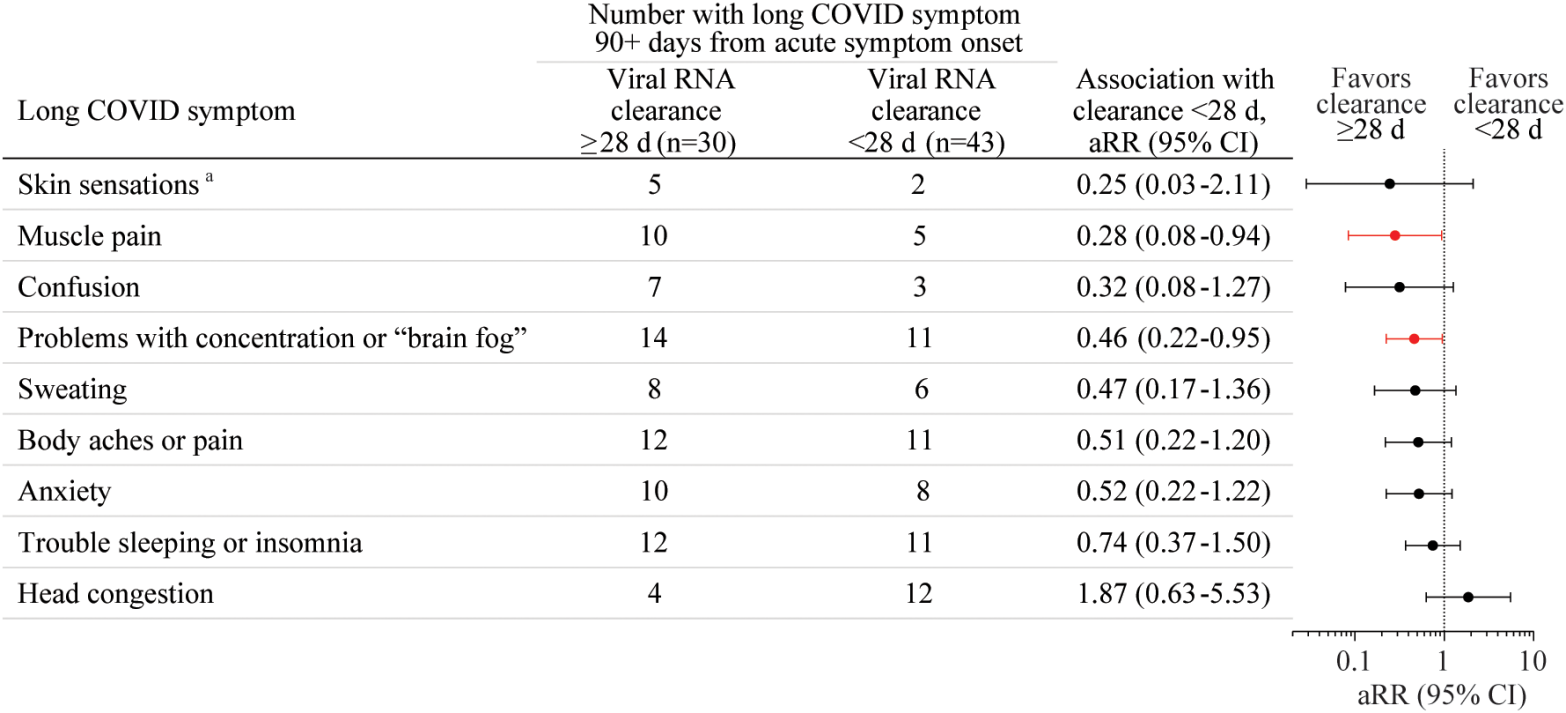
The association of the presence of each of the 49 long coronavirus disease (COVID) symptoms at 90+ days after acute COVID-19 illness onset with SARS-CoV-2 RNA clearance from the upper respiratory tract (URT, mid-turbinate nasal and/or saliva) within 28 days from acute COVID-19 onset was tested without adjustment using log-binomial regression analyses. Nine long COVID symptoms with *p*-values <0.2 were tested using log-binomial regression analyses with adjustment for age, sex, BMI over/under 25, and COVID vaccination status prior to acute COVID-19 (1+ vaccines *vs*. none), with results shown here. aRR, adjusted risk ratio, d, day. ^a^Tingling, numbness, coldness, or other unusual sensation in the skin.

The association of the presence of each of the 49 long COVID symptoms (**Table 1**) at 90+ days post-acute COVID-19 onset with time to SARS-CoV-2 RNA clearance from the URT within 28 days was first tested without adjustment using log-binomial regression analyses. Eleven symptoms with *p*-values <0.2 were tested using log-binomial regression analyses with adjustment for age, sex, BMI ≥25, and COVID vaccination status prior to acute COVID-19. Brain fog and muscle pain at 90+ days after acute COVID-19 illness onset were negatively associated with viral RNA clearance within 28 days with these adjustments [**Figure 1**, brain fog: adjusted risk ratio (aRR) 0.46, 95% CI 0.22-0.95; muscle pain: aRR 0.28, 95% CI 0.08-0.94]. The model failed to converge for two rarely reported symptoms: leg swelling and problems with balance. Leg swelling at 90+ days from acute COVID-19 onset was negatively associated with viral RNA clearance within 28 days of acute COVID-19 onset in an unadjusted analysis (RR 0.17, 95% CI 0.04-0.76) and in an analysis adjusted only for age and sex (aRR 0.21, 95% CI 0.05-0.82). Problems with balance at 90+ days from acute COVID-19 onset were negatively associated with viral RNA clearance within 28 days of acute COVID-19 onset only in an analysis adjusted for age, sex, and vaccination status (aRR 0.47, 95% CI 0.25-0.90) but not in a univariate analysis. There were non-significant trends toward negative associations of body aches or pain, anxiety, confusion, and sweating with viral RNA clearance within 28 days after adjustment for age, sex, BMI ≥25, and COVID vaccination status prior to acute COVID-19 (**Figure 1**). No other associations of long COVID symptoms with viral RNA clearance time within 28 days reached statistical significance.

The acute viral RNA decay trajectories of participants who did and did not later go on to experience brain fog 90 or more days from acute COVID-19 illness onset were distinct (**Supplementary Figure 2**). Participants reporting higher severity brain fog or muscle pain at 90 or more days from acute COVID-19 illness onset were less likely to have cleared SARS-CoV-2 RNA within 28 days. In logistic regression models adjusted for age, sex, and BMI ≥25, with each 1-level increase of maximum reported brain fog or muscle pain on a scale of 1-5, the odds of clearance in 28 days decreased (brain fog: aOR 0.58, 95% CI 0.36-0.94; muscle pain: aOR 0.44, 95% CI 0.26-0.76).

## 4 Discussion

We demonstrate here that post-acute brain fog and muscle pain are associated with prolonged time to clearance of SARS-CoV-2 RNA from the upper respiratory tract during acute COVID-19. This finding provides evidence that delayed immune clearance of SARS-CoV-2 antigen and/or greater amount or duration of viral antigen burden in the upper respiratory tract during acute COVID-19 is directly linked to long COVID. This work indicates that host–pathogen interactions during the first few weeks after COVID-19 symptom onset have an impact on long COVID risk months later.

Currently, the biological mechanisms underlying long COVID and its myriad symptoms are unclear. There are several different hypotheses: persistent immune dysregulation following acute COVID-19, persistent SARS-CoV-2 virions or antigen (“reservoirs”) triggering chronic inflammation, reactivation of other viruses such as the Epstein–Barr virus (EBV), autoimmunity triggered by acute illness, endothelial dysfunction and/or microthrombi impairing organ function, metabolic or mitochondrial dysfunction on the cellular level, autonomic nervous system injury, lingering or evolving organ injury from acute COVID-19, and microbiota alterations, among others (11). A recent comprehensive autopsy study provided evidence for an early viremic phase during acute COVID-19 that spreads infectious virus throughout the body and demonstrated convincingly that SARS-CoV-2 RNA can persist for >30 days in multiple extrapulmonary sites (12).

There have been a few studies that link long COVID with persistent SARS-CoV-2 antigen detected in the late post-acute time period, notably spike protein in plasma (13), SARS-CoV-2 proteins in plasma neuron- and astrocyte-derived extracellular vesicles (14), spike protein in monocytes (15), and fecal RNA shedding (16). However, there has been just one study to our knowledge linking virus kinetics or detection during acute COVID-19 with long COVID; this study associated the presence or absence of SARS-CoV-2 RNA in the plasma at the time of COVID-19 diagnosis and hospitalization with one or more symptoms of long COVID several months later (17). However, this study was limited to people with severe acute COVID-19 (17), and it may be that the presence of SARS-CoV-2 RNAemia at hospital presentation is an indicator of the severity of acute COVID-19, which is already known to be a risk factor for long COVID (1).

Our study provides robust support for the hypothesis that viral kinetics during the acute phase of illness is associated with long COVID by using frequent longitudinal sampling beginning within days of acute COVID-19 onset to determine the duration of viral RNA shedding from the URT. Additionally, since long COVID may comprise one or more syndromes with unique mechanisms, we assessed individual symptoms from multiple organs or domains affected by long COVID to determine whether particular syndromes or symptom clusters of long COVID are associated with viral kinetics in the acute phase. We studied a population that primarily had mild–moderate COVID-19 and, thus, is more representative of the general population of people who had COVID-19 or long COVID and limits the amount by which the severity of acute illness could confound our results. A final strength of this cohort is the racial and ethnic diversity of participants. A weakness of the study is that the intensity of study coordination involved in repeated longitudinal sampling and follow-up in acute and post-acute COVID-19 limited the number of participants enrolled.

Our data are consistent with a few biologically plausible scenarios. First, both delayed clearance of viral antigen and long COVID are caused by another factor such as dysregulated or distracted immunity. This dysregulated immunity might cause long COVID by reactivating other latent viruses, triggering autoimmunity, causing direct organ damage, or delaying tissue repair from acute infection. Second, persistent expression of viral antigens, and/or a larger burden of initial antigen, might directly cause long COVID by sustaining tissue injury and inflammation. The latter mechanism is supported by early studies linking antiviral treatment of people during acute COVID-19 with reduced incidence of long COVID (18). More studies with longitudinal repeated sampling beginning from acute COVID-19 illness treated with and without antivirals through the post-acute phase are urgently needed to assess how host–pathogen interactions lead to long COVID.

Here, we report that at least two long COVID symptoms—brain fog and muscle pain—at 90+ days from acute COVID-19 onset are specifically associated with longer time to clearance of SARS-CoV-2 RNA from the upper respiratory tract during acute illness.

## Data availability statement

The raw data supporting the conclusions of this article will be made available upon request, without undue reservation.

## Ethics statement

The studies involving human participants were reviewed and approved by the Johns Hopkins University School of Medicine IRB. Written informed consent for participation was not required for this study in accordance with the national legislation and the institutional requirements.

## Author contributions

AA, TY, and YM: conception and design. AA, TY, and CH: statistical analysis and interpretation of data. PB and YM: funding acquisition. DT and YM: administrative, technical, or material support. All authors: writing, review, and critical revision of the manuscript for important intellectual content. AA and YM: study supervision. All authors contributed to the article and approved the submitted version.

## OutSMART Study Group

M. Gabriela Varela Heslin, Sarika K. Mullapudi, Chamia Dorsey, Christine Payton, Sidney-Saint-Hilaire, Zihan Yang, Justin Chan, Razvan Azamfirei, Minyoung Jang, Taylor Church, Carolyn Reuland, Vismaya S. Bachu, Jennifer L. Townsend, Sara C. Keller, Jeanne C. Keruly, Justin P. Hardick, Madison Conte, and Thelio Sewell.
