# Supplementary material for: Long COVID brain fog and muscle pain are associated with longer time to clearance of SARS-CoV-2 RNA from the upper respiratory tract during acute infection
Source: Front Immunol. 2023 Apr 28;14:1147549. doi: 10.3389/fimmu.2023.1147549 (PMC10176965; doi:10.3389/fimmu.2023.1147549)
Supplement: Supplementary file 1 [file DataSheet_1.docx]

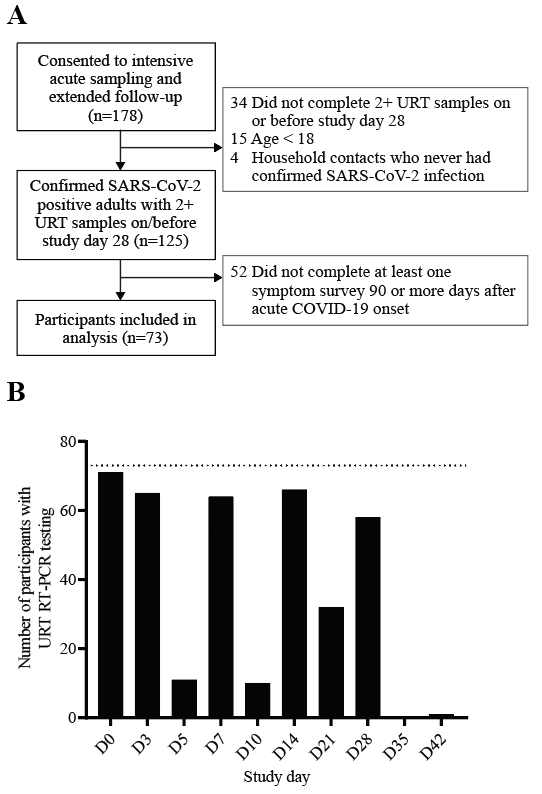


**Supplementary Figure 1.** Participant eligibility flow diagram and upper respiratory tract sampling in the first 42 study days. (A) A convenience sample of 1,086 adults with recent positive SARS-CoV-2 RT-PCR tests from outpatient testing sites of the Johns Hopkins Health System was assessed for eligibility between April 21, 2020, and October 28, 2021. Between December 9, 2020, and October 28, 2021, 48 adult or child household contacts of eligible participants were contacted with their permission to assess eligibility for enrollment in the study. Of these, 178 people consented to a protocol of intensive early upper respiratory tract sampling and follow-up through 24 months post-enrollment. (B) Among the 73 participants who met criteria for inclusion in this analysis, the number who completed upper respiratory tract sampling for SARS-CoV-2 RT-PCR testing on each study day is depicted. Study day sampling on days 5, 10, 21, 35, and 42 were not required for all participants. Most study days included sampling of both midturbinate nasal samples and oral samples (saliva, oropharyngeal, or gingival crevicular fluid) but occasionally just one type is available. The horizonal dashed line is placed at 73. URT, upper respiratory tract; RT-PCR, reverse transcription polymerase chain reaction.


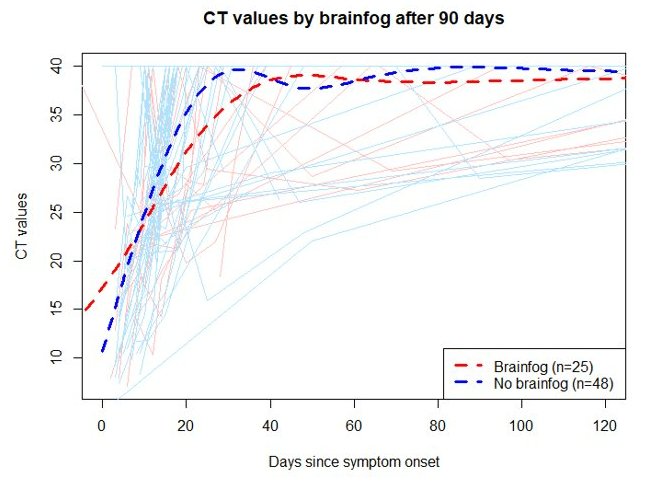


**Supplementary Figure 2.** Functional data analysis methods were used to generate estimated mean functions of SARS-CoV-2 RT-PCR cycle threshold (CT) values from mid-turbinate nasal samples by day from acute COVID-19 onset in individuals who later reported brain fog/problems with concentration at 90 or more days from acute COVID-19 onset (red) and those who reported no brain fog/problems with concentration at 90 or more days from acute COVID-19 onset (blue).
